# Supplementary material for: Zika virus RNA structure controls its unique neurotropism by bipartite binding to Musashi-1
Source: Nat Commun. 2023 Feb 28;14:1134. doi: 10.1038/s41467-023-36838-w (PMC9972320; doi:10.1038/s41467-023-36838-w)
Supplement: Supplementary file 1 — Supplementary Information [file 41467_2023_36838_MOESM1_ESM.pdf]

Supplementary Information for

Zika virus RNA structure controls its unique neurotropism by bipartite binding to Musashi-1

Xiang Chen[1, #], Yan Wang[2, #], Zhonghe Xu[2, #] Meng-Li Cheng[1], Qing-Qing Ma[1], Rui-Ting Li[1], Zheng-Jian Wang[1], Hui Zhao[1], Xiaobing Zuo[3], Xiao-Feng Li[1], Xianyang Fang[2, 4, *], Cheng-Feng Qin[1, 5, *]

Correspondence to: Prof. Cheng-Feng Qin (Email: qincf@bmi.ac.cn) or Prof. Xianyang Fang (Email: fangxy@ibp.ac.cn)

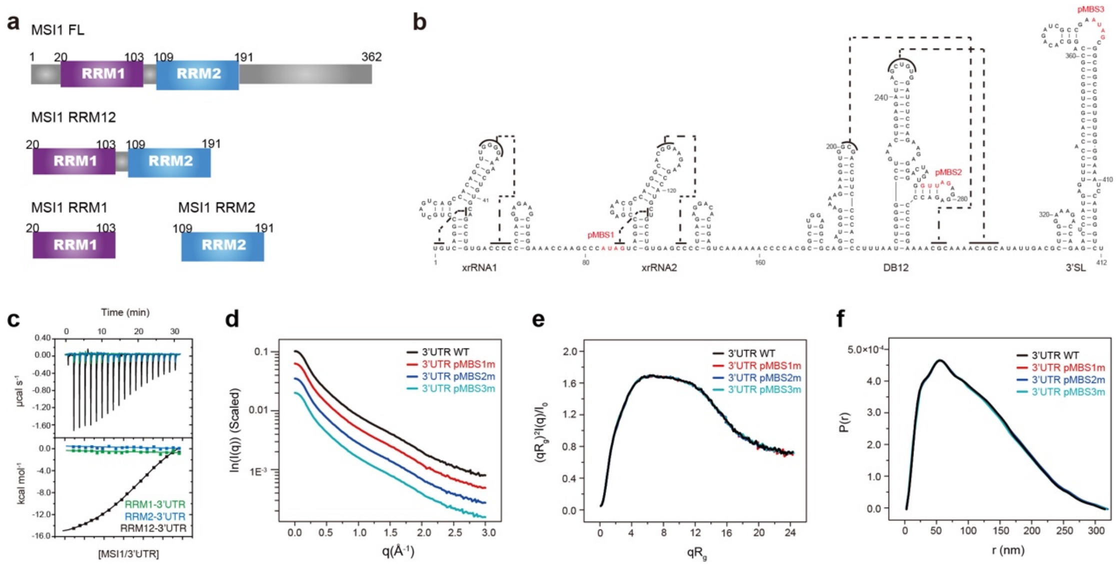

Fig. S1. Structure and interaction of human Musashi-1 and ZIKV 3’UTR. related to Fig.1. (**a**) Schematic representation of domain organization of human Musashi-1 and constructs of its subdomains (RRM12, RRM1 and RRM2) used in this study. (**b**) Secondary structure of the ZIKV 3’UTR. The individual putative MSI1 binding sites (pMBSs, pMBS1-3) are dedicated. (**c**) The ITC profiles of RRM1, RRM2 and RRM12 binding to ZIKV 3’UTR. (**d-f**) Structural characterization of pMBS mutants of ZIKV 3’UTR by SAXS. SAXS profiles (d), the dimensionless Kratky plots (e) and paired distance distribution functions (PDDFs) transformed from the scattering profiles (f) of the WT (black), pMBS1m (red), pMBS2m (blue) and pMBS3m (cyan) mutants of ZIKV 3’UTR.

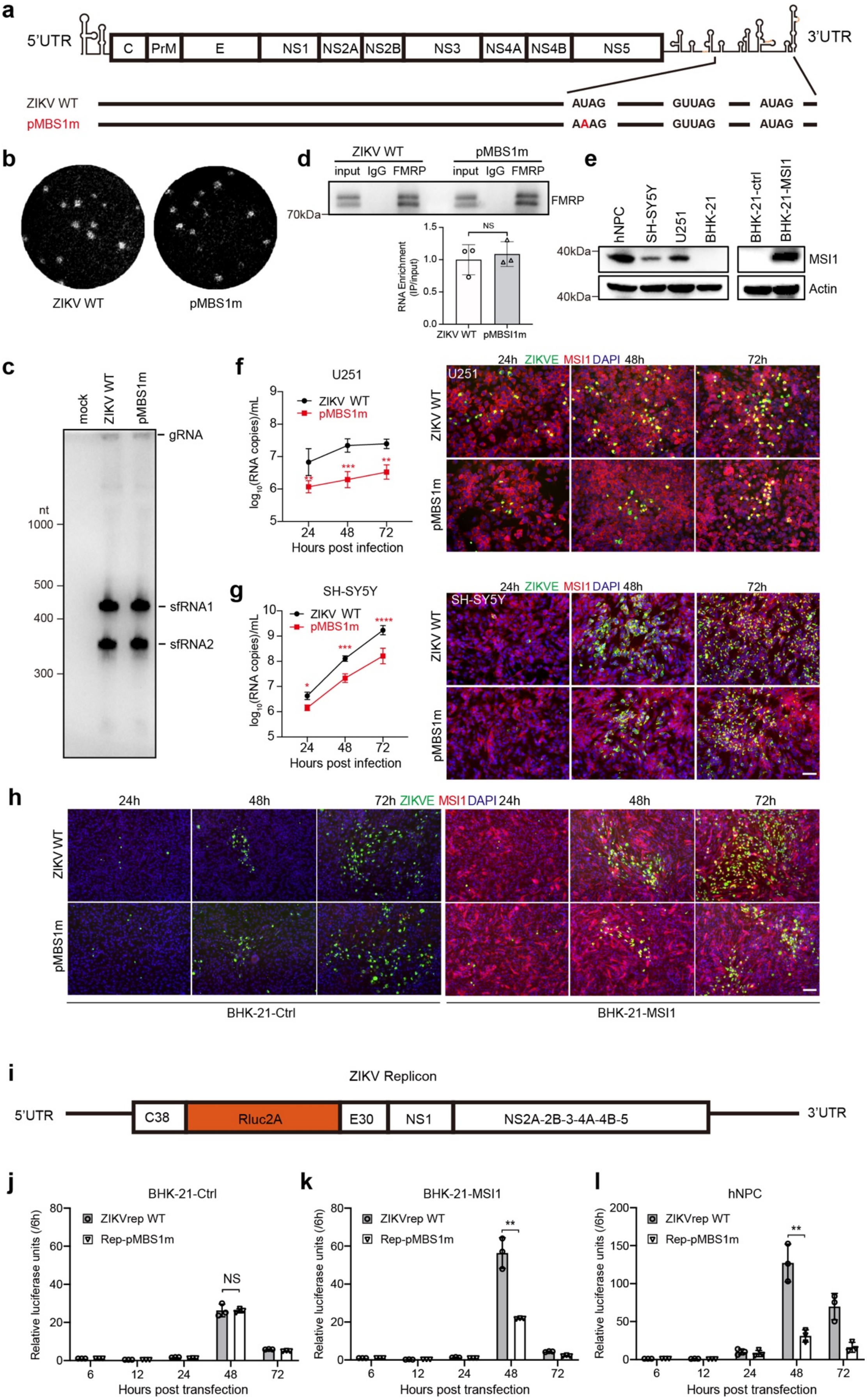
a
5'UTR
C
PrM
E
NS1
NS2A
NS2B
NS3
NS4A
NS4B
NS5
3'UTR
ZIKV WT
pMBS1m
AUAG
GUUAG
AUAG
AAAG
GUUAG
AUAG
b
ZIKV WT
pMBS1m
c
mock
ZIKV WT
pMBS1m
gRNA
nt
1000
500
400
300
sfRNA1
sfRNA2
d
ZIKV WT
pMBS1m
input
IgG
FMRP
70kDa
FMRP
NS
RNA Enrichment (IP/input)
1.5
1.0
0.5
0.0
ZIKV WT
pMBSI1m
e
hNPC
SH-SY5Y
U251
BHK-21
BHK-21-ctrl
BHK-21-MSI1
40kDa
MSI1
40kDa
Actin
f
U251
ZIKV WT
pMBS1m
log10(RNA copies)/mL
Hours post infection
24h ZIKVE MSI1 DAPI 48h
72h
g
SH-SY5Y
ZIKV WT
pMBS1m
log10(RNA copies)/mL
Hours post infection
24h ZIKVE MSI1 DAPI 48h
72h
h
24h
48h
72h ZIKVE MSI1 DAPI 24h
48h
72h
ZIKV WT
pMBS1m
BHK-21-Ctrl
BHK-21-MSI1
i
ZIKV Replicon
5'UTR
C38
Rluc2A
E30
NS1
NS2A-2B-3-4A-4B-5
3'UTR
j
BHK-21-Ctrl
ZIKVrep WT
Rep-pMBS1m
NS
Relative luciferase units (/6h)
Hours post transfection
k
BHK-21-MSI1
ZIKVrep WT
Rep-pMBS1m
Relative luciferase units (/6h)
Hours post transfection
l
hNPC
ZIKVrep WT
Rep-pMBS1m
Relative luciferase units (/6h)
Hours post transfection

Fig. S2. The pMBS1 mutation attenuated ZIKV replication in cells with MSI1 expression. related to Fig.2. (**a**) Schematics of construction of ZIKV WT and corresponding 3'UTR mutant viruses. (**b**) Plaque morphology of ZIKV WT and pMBS1m. (**c**) BHK-21 cells were mock infected or infected with WT or pMBS1m. 48 hours post-infection, cell-associated RNA was harvested and levels of gRNA and sfRNA were analyzed by Northern Blot. Similar result was repeated independently in 2 times. (**d**) RNA-IP analysis from WT or pMBS1m infected BHK-21 cells. Western blot shows immunoprecipitations (IPs) by immunoglobulin G (IgG) and FMRP antibodies. Input (5%) represents whole-cell extract. Western blot was probed with antibodies against FMRP. Graph below shows quantitative polymerase chain reaction (qPCR) performed on bound RNA from IP. RNA-IP values are presented as the ratio to the input after subtraction of the IgG background. Data are mean ±SD. n=3 independent experiments. P values were obtained from two-sided Student's *t* test, NS, not significant. (**e**) MSI1 protein expression from hNPC, SH-SY5Y, U251, BHK-21, BHK-21-ctrl and BHK-21-MSI1 cells were detected by Western blot. Similar result was repeated independently in 2 times. (**f-g**) Viral RNA copies in culture supernatants and viral envelope protein expression of U251 (f) and SH-SY5Y (g) cells at the indicated time points after infected with ZIKV WT and pMBS1m (U251: MOI=1; SH-SY5Y: MOI=0.1). ZIKV E(green), MSI1(red). Scale bar, 50um. Data are mean ±SD. n=3 independent experiments. Two-way ANOVA, *P<0.05, **P<0.01, ***P<0.001, ****P<0.0001. (f: 24h P=0.0092; 48h P=0.0008; 72h P=0.0035. g: 24h P=0.0254; 48h P=0.0007; 72h P<0.0001). (**h**) WT and pMBS1m viral E protein expression in BHK-21-Ctrl and BHK-21-MSI1 cells, related to Fig. 2c. ZIKV E(green), MSI1(red). Scale bar, 50um. (**i**) Schematic of ZIKV luciferase replicon. Similar result was repeated independently in 3 times. (**j-l**) Replication kinetics of the wild type and mutated ZIKV replicon Rep-pMBS1m in transfected BHK-21 cells (j), BHK-21-MSI1 cells (k) and hNPC cells (l). The ratios of raw luciferase units measured at different time points after transfection to the value measured at 6 hours post transfection (relative luciferase units) are shown. Data are mean ± SD. n=3 independent experiments. Two-sided Student's *t* test, **P < 0.01, NS, not significant. (j: P=0.0017; l: P=0.0031). Source data are provided as a Source Data file.

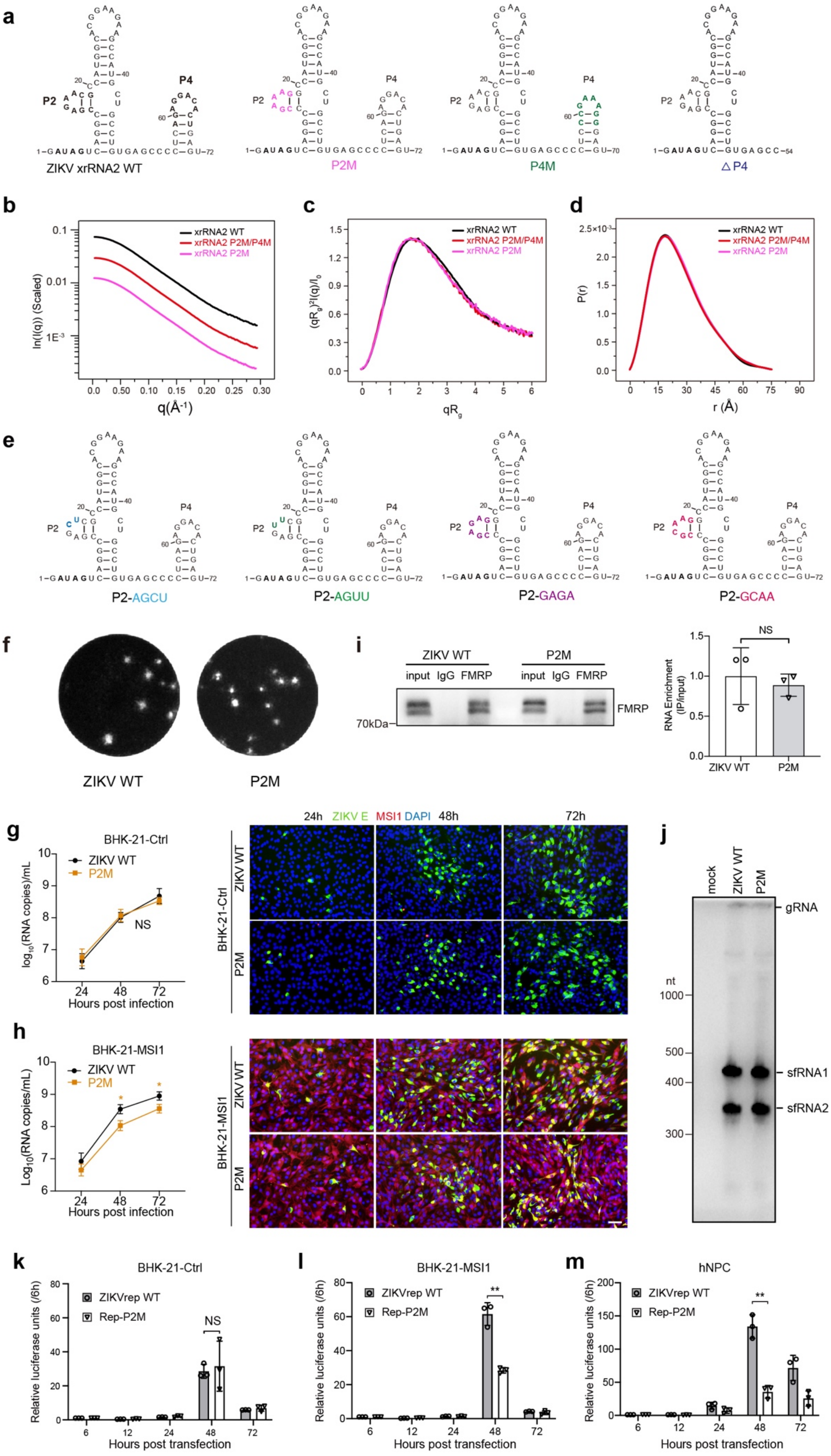
a
P2
P4
ZIKV xrRNA2 WT
P2M
P4M
△P4
b
xrRNA2 WT
xrRNA2 P2M/P4M
xrRNA2 P2M
ln(I(q)) (Scaled)
q(Å-1)
c
(qRg)²I(q)/I0
qRg
d
P(r)
r (Å)
e
P2-AGCU
P2-AGUU
P2-GAGA
P2-GCAA
f
ZIKV WT
P2M
i
ZIKV WT
P2M
input IgG FMRP
70kDa
FMRP
RNA Enrichment (IP/input)
NS
g
BHK-21-Ctrl
ZIKV WT
P2M
log10(RNA copies)/mL
NS
Hours post infection
24h
ZIKV E
MSI1
DAPI
48h
72h
h
BHK-21-MSI1
Log10(RNA copies/mL)
j
mock
gRNA
nt
sfRNA1
sfRNA2
k
Relative luciferase units (/6h)
ZIKVrep WT
Rep-P2M
Hours post transfection
l
m
hNPC

Fig. S3. The AGAA tetraloop capping P2 stem of ZIKV xrRNA2 and its fold are crucial to MSI1 binding. related to Fig.3 and Fig.4. (**a)** Secondary structure of xrRNA2 and its mutant constructs. P2M: only the P2 loop was mutated to GAAA tetraloop; P4M: only the P4 loop was mutated to GAAA tetraloop; ΔP4: the total P4 stem-loop was deleted. (**b-d)** Structural characterization of xrRNA2 mutants by SAXS. SAXS profiles (b), the dimensionless Kratky plots (c) and paired distance distribution functions (PDDFs) (d) of xrRNA2 WT, mutants of P2M/P4M and P2M. **(e)** Secondary structures of xrRNA2 constructs. Mutants with AGNN-type tetraloop include P2-AGCU and P2-AGUU. Mutants with GNRA-type tetraloop include P2-GAGA and P2-GCAA. (**f**) Plaque morphology of ZIKV WT and P2M. (**g-h**) Viral RNA copies in culture supernatants and viral envelope protein expression of BHK-21-Ctrl cells (g) and BHK-21-MSI1 cells (h) at the indicated time points after ZIKV WT and pMBS1M infection (MOI=0.01). Data are mean ±SD. n=3 independent experiments. Two-way ANOVA was performed for statistical analysis. *P<0.05, NS not significant. (h: 48h P=0.0103; 72h P=0.0476). ZIKV E (green), MSI1 (red). Scale bar, 50um. (**i**) RNA-IP analysis from WT or P2M infected BHK-21 cells. Western blot shows immunoprecipitations (IPs) by immunoglobulin G (IgG) and FMRP antibodies. Input (5%) represents whole-cell extract. Western blot was probed with antibodies against FMRP. Graph below shows quantitative polymerase chain reaction (qPCR) performed on bound RNA from IP. RNA-IP values are presented as the ratio to the input after subtraction of the IgG background. Data are mean ±SD. n=3 independent experiments. P values were obtained from Student's *t* test, NS, not significant. (**j**) BHK-21 cells were mock infected or infected with WT or P2M. 48 hours post-infection, cell-associated RNA was harvested and levels of gRNA and sfRNA were analyzed by Northern Blot. Similar result was repeated independently in 2 times. (**k-m**) Replication kinetics of the wild type and mutated ZIKV replicon Rep-P2M in transfected BHK-21-Ctrl cells (k), BHK-21-MSI1 cells (l) and hNPCs (m). The ratios of raw luciferase units measured at different time points after transfection to the value measured at 6 h post transfection (relative luciferase units) are shown. Data are mean ± SD. n=3 independent experiments. Two-sided Student's *t* test, *P<0.05, **P<0.01, NS not significant. (l: P=0.0011. m: 48h P=0.0011; 72h P=0.0225). Source data are provided as a Source Data file.

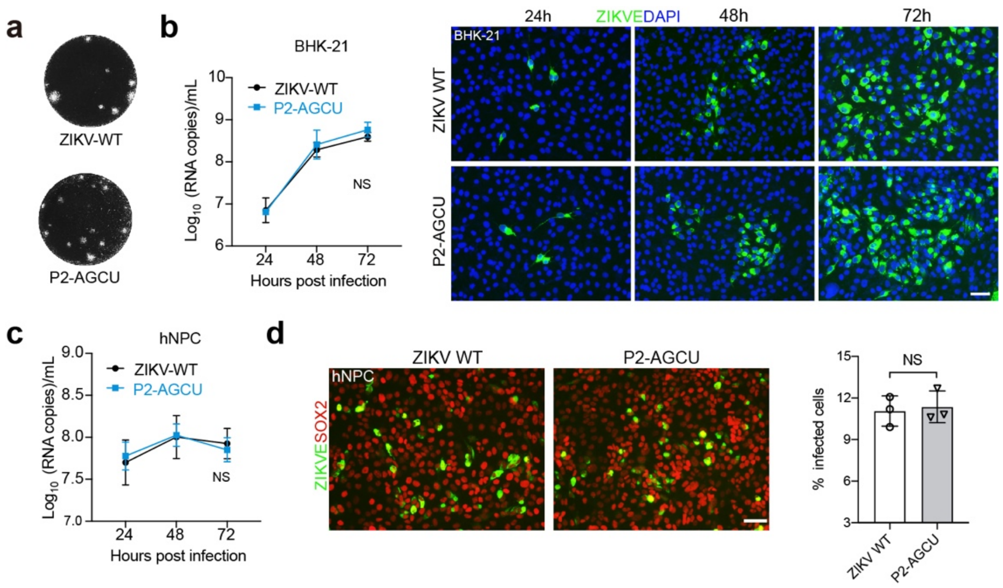

Fig. S4. The P2-AGCU mutation does not affect MSI1 binding or ZIKV replication. (**a**) Plaque morphology of ZIKV WT and P2-AGCU. (**b**) Viral RNA copies in culture supernatants and viral envelope protein expression of BHK-21 cells at the indicated time points after infected with the ZIKV WT or P2-AGCU viruses (MOI=0.01). Scale bar, 50 µm. Data are mean ±SD. n = 3 independent experiments. Two-way ANOVA, NS not significant. (**c**) hNPCs were infected with the ZIKV WT or P2-AGCU viruses (MOI=1), and the culture supernatants harvested at the indicated time points for detection of viral RNA copies by qRT-PCR. Data are mean ±SD. n = 3 independent experiments. Two-way ANOVA, NS not significant. (**d**) The expression of viral envelope protein at 48 hours after infection from (c) was detected by immunostaining. Scale bar, 50 µm. Data are Mean ±SD. Two-sided Student's *t* test, NS not significant. Source data are provided as a Source Data file.

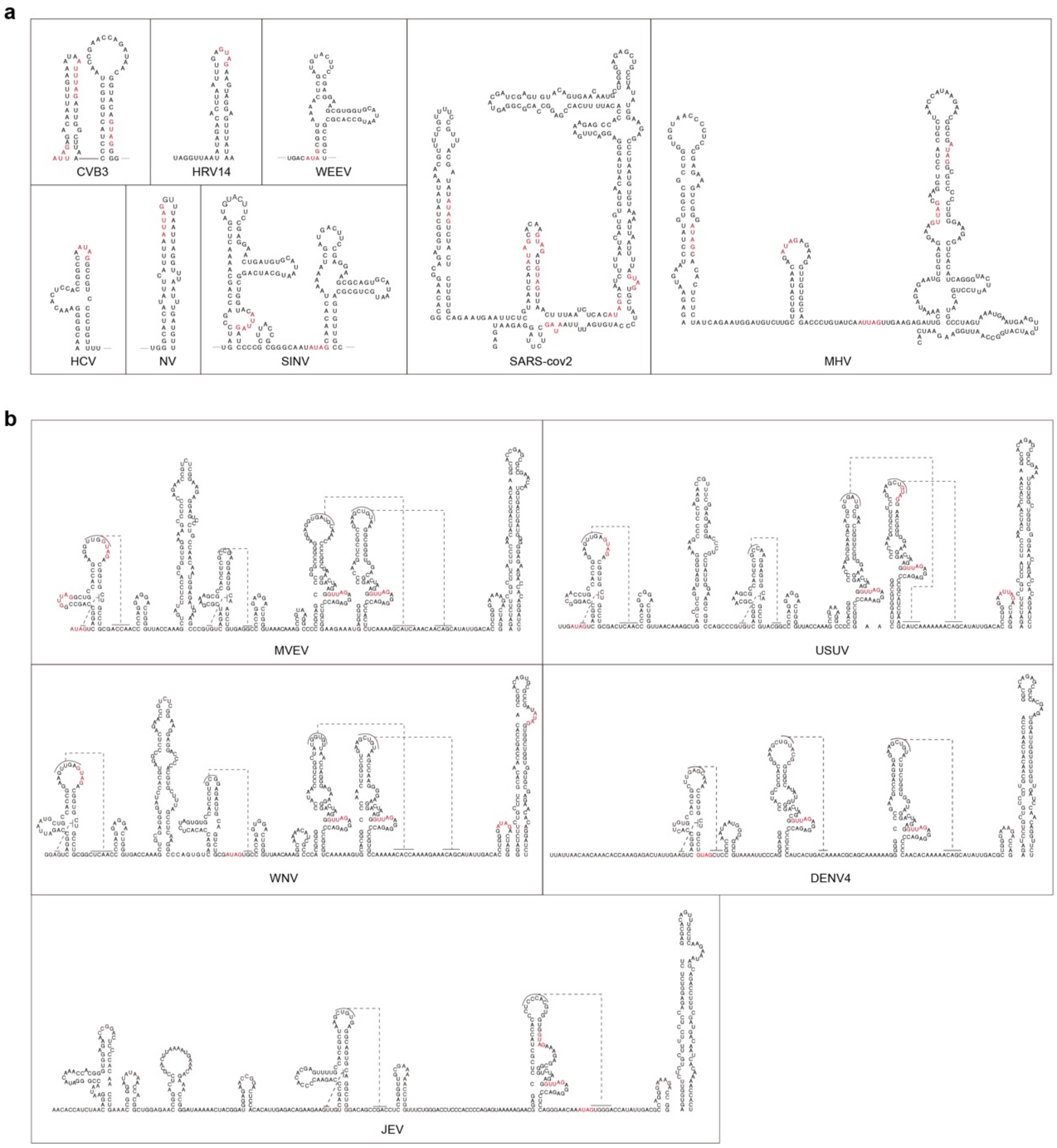

Fig. S5. Secondary structures of the 3'UTRs of viruses that harbor the pMBS (G/A$_{(1-3)}$UAG) sequences (in red). related to Fig.5. (**a**) These viruses include: CVB3: coxsackievirus B3; HCV: Hepatitis C Virus; HRV14: human rhinovirus 14; NV: Norwalk virus; WEEV: western equine encephalitis virus; SINV: sindbis virus; SARS-Cov2: severe acute respiratory syndrome coronavirus 2; MHV: Murine hepatitis virus. (**b**) Secondary structure prediction of other MBFV 3'UTRs using RNAfold and RNAalifold. The pMBSs (G/AU$_{(1-3)}$AG) are colored with red. Pseudoknots are indicated with black lines.

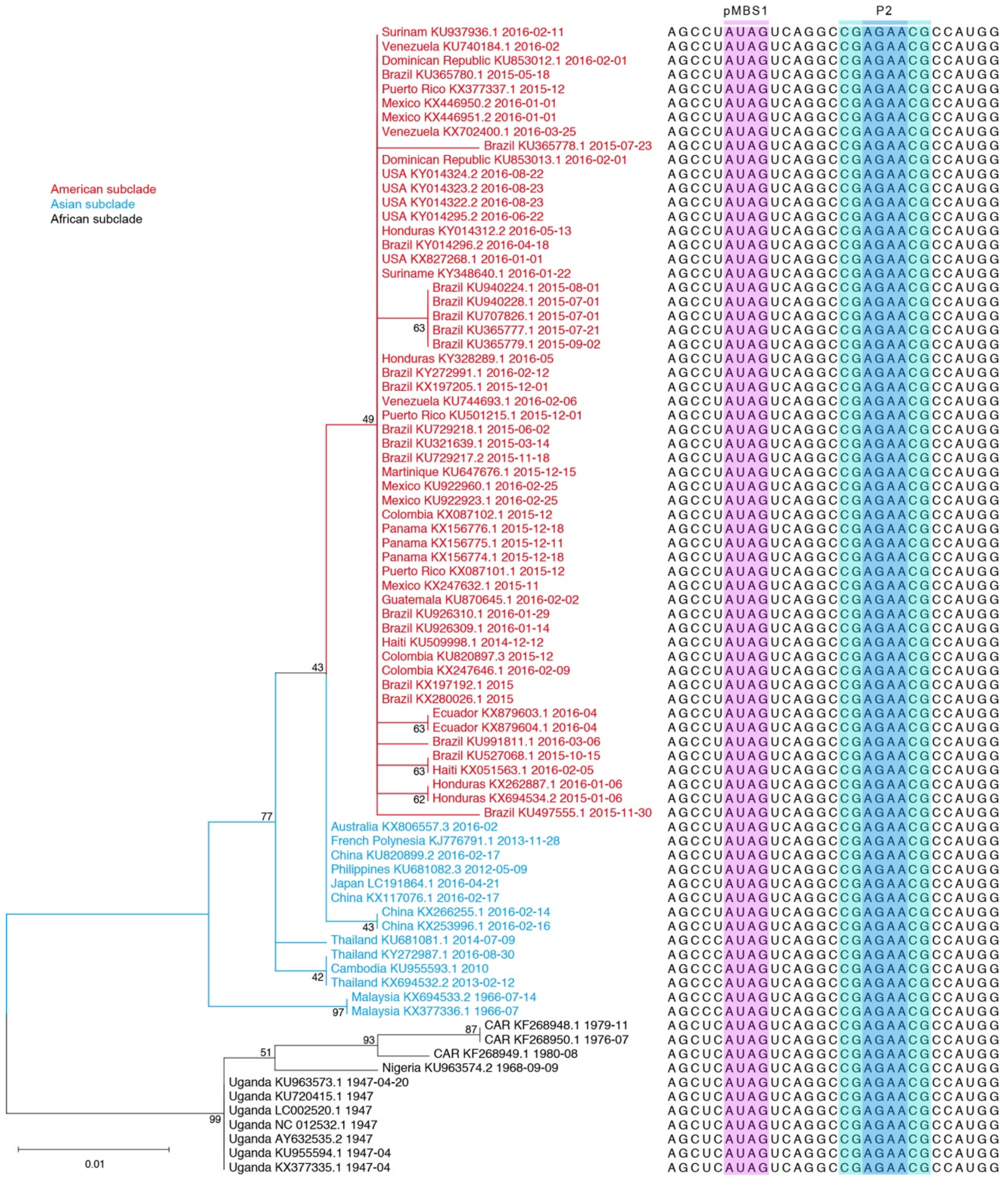

Fig. S6. The two MBSs of xrRNA2 are conserved in ZIKV isolates. related to Fig.5. A maximum likelihood tree was constructed with the 3'UTR sequences of ZIKVs by using MEGA. The pMBS1 and P2 sequences (colored as in Fig. 5a) of xrRNA2 were shown after the virus IDs to highlight the conserved AUAG and P2 elements with the phylogenetic clades.

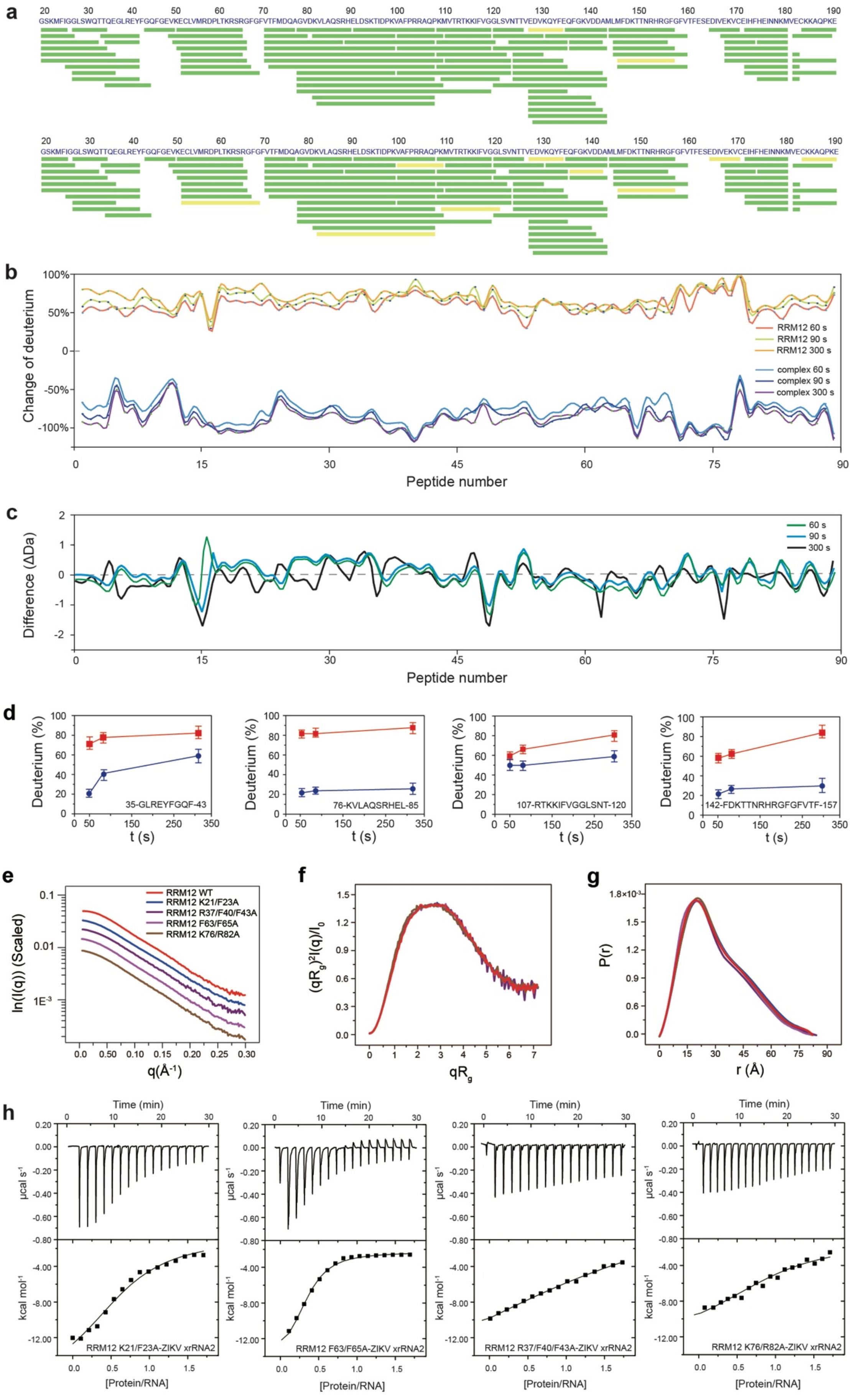

a
b
Change of deuterium
Peptide number
RRM12 60 s
RRM12 90 s
RRM12 300 s
complex 60 s
complex 90 s
complex 300 s
c
Difference (ΔDa)
Peptide number
60 s
90 s
300 s
d
Deuterium (%)
t (s)
35-GLREYFGQF-43
76-KVLAQSRHEL-85
107-RTKKIFVGGLSNT-120
142-FDKTTNRHRGFGFVTF-157
e
ln(I(q)) (Scaled)
q(Å-1)
RRM12 WT
RRM12 K21/F23A
RRM12 R37/F40/F43A
RRM12 F63/F65A
RRM12 K76/R82A
f
(qRg)²I(q)/I0
qRg
g
P(r)
r (Å)
h
Time (min)
µcal s-1
kcal mol-1
[Protein/RNA]
RRM12 K21/F23A-ZIKV xrRNA2
RRM12 F63/F65A-ZIKV xrRNA2
RRM12 R37/F40/F43A-ZIKV xrRNA2
RRM12 K76/R82A-ZIKV xrRNA2

Fig. S7. Mapping the RNA binding interfaces within MSI1 RRM12 using the HDX-MS technique. related to Fig.7. (**a**) Sequence coverage maps of RRM12 alone (*apo*-form, top) and in the presence of 2-fold xrRNA2 (*holo*-form, bottom). Peptides with high confidence and medium confidence are colored with green and yellow, respectively. (**b**) The relative deuterium uptake plots of RRM12 alone and in complex of xrRNA2. (**c**) Averaged differential plots of deuterium uptake of peptides at time course of 60s, 90s and 300 s. (**d**) Deuterium uptake curves for the four peptides showing significant differences between *apo*-form (blue) and *holo*-form (red) states. Data are mean ± SD. n = 3 independent experiments. (**e-g**) The SAXS scattering profiles (e), the dimensionless Kratky plots (f) and the normalized PDDFs (g) of RRM12 wild type, mutants of RRM12 K21/F23A, RRM12 R37/F40/F43A, RRM12 K76/R82A and RRM12 F63/F65A. (**h**) ITC profiles of mutants of RRM12 K21/F23A, R37/F40/F43A, K76/R82A and F64/F65A to ZIKV xrRNA2 wild type.

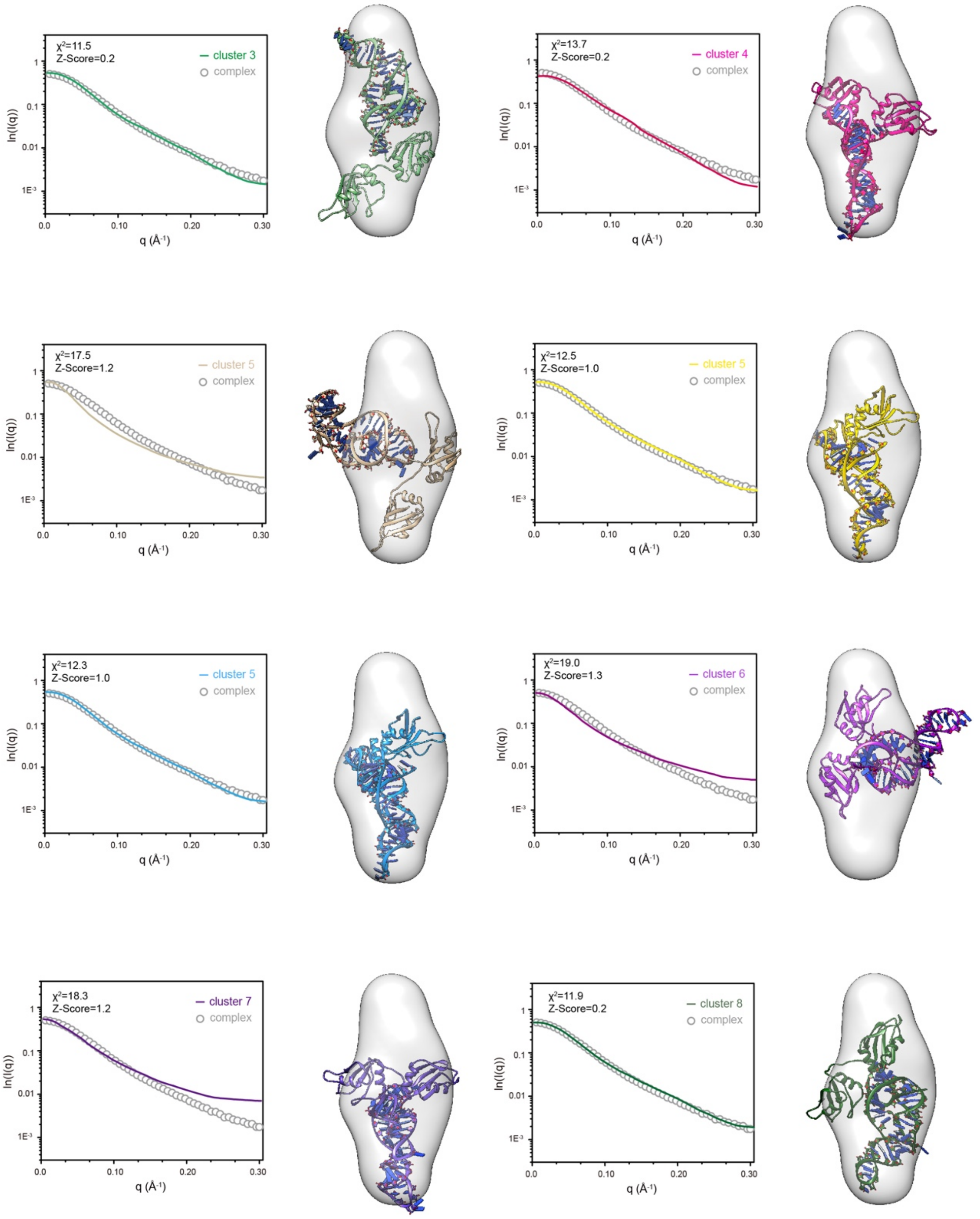

Fig. S8. Structural validation of the representative models from clusters 3-10. The theoretical scattering curves of the representative models from the respective clusters (solid lines) were overlaid with the experimental SAXS data of RRM12-xrRNA2 complex (open circle). The representative models are fitted into the 3D shape envelope *ab initio* reconstructed by DAMMIN. The HADDOCK Z-scores and the fitting χ^2 are included in the respective panels.

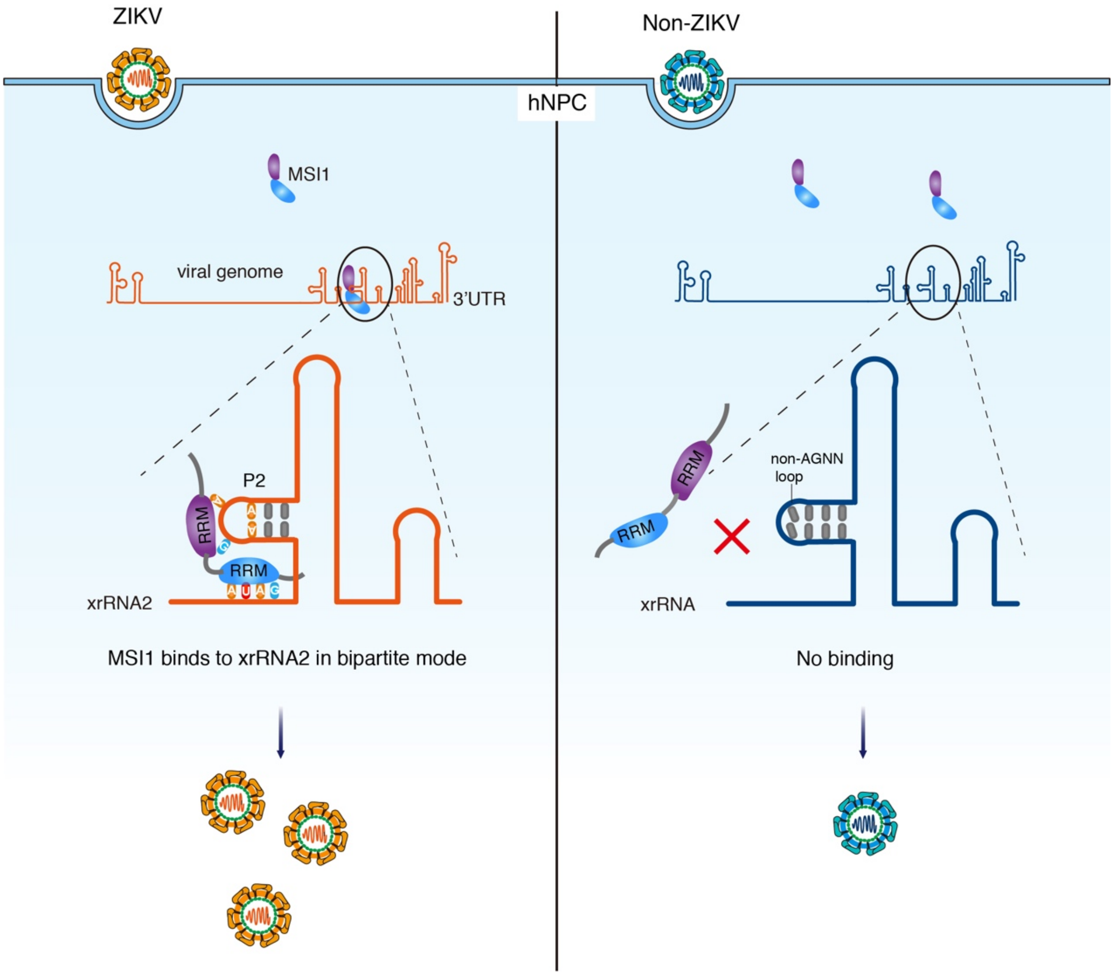

Fig. S9. Model for the mechanism by which ZIKV xrRNA2 binds to MSI1 and promotes viral replication. Upon ZIKV infection in cells expressing MSI1, ZIKV utilize a sequence-specific motif (pMBS1) and an unusual structure-specific motif (P2) to respectively bind to two RRMs of MSI1. This bipartite binding mode renders ZIKV RNA a high affinity to MSI1 and promotes viral replication. However, other flaviviruses do not possess both MBSs in their 3'UTRs like ZIKV does, so they are unable to bind MSI1 and consequently cannot exploit MSI1 to replicate.

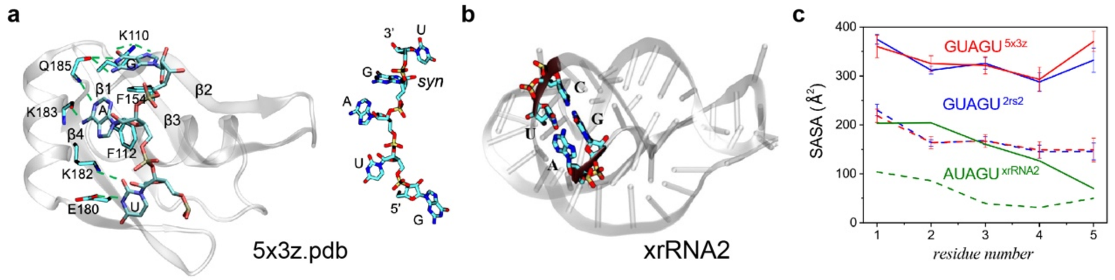

Fig. S10. Structural features of single-stranded UAG trinucleotide in complex with RRM2 and base-paired AG dinucleotide within xrRNA2. related to Fig.7. (**a**) Canonical recognition of UAG trinucleotide by RRM2 (left). For clarity, the nucleotides preceding and following UAG are not shown. The hydrogen bonds between RRM2 and **UAG** are shown in bond. Zoom-in view of G**UAG**U(right). In the Binding-Mode 1, the interaction of pMBS1 with RRM2 is similar to the canonical feature. (**b**) The base pairs between **AG** (part of AU**AG**U) and CU (J31) which form PK1 are embedded in the core of xrRNA2 and shown in bond. (**c**) Comparison of the solvent accessible surface area (SASA) for GUAGU from 2RS2.pdb (RRM1) and 5X3Z.pdb, and AUAGU from xRNA2. The SASA for nucleotide and nucleobase are plotted as solid or dash line, respectively. Data are mean ± SD. n = 20 structure models from NMR ensemble.

Table S1. Basic structural parameters for ZIKV xrRNA2 and its mutants, ZIKV xrRNA1, human MSI1 RRM12 and its mutants, and the xrRNA2-RRM12 complex by SAXS.

Sample	R_g(Å)[a]	R_g(Å)[b]	D_{max}(Å)	MW[c]	MW[d]
xrRNA2 WT	22.0 ± 0.2	22.6 ± 0.3	83	23.4	21.7
xrRNA2 pMBS1m	22.5 ± 0.6	23.0 ± 0.7	84	23.4	22.0
xrRNA2 P2M	22.3 ± 0.1	22.9 ± 0.1	83	23.4	22.0
xrRNA2 P2/P4M	22.1 ± 0.1	22.8 ± 0.2	83	23.4	22.1
xrRNA2-ΔPK1	29.0 ± 0.5	29.3 ± 0.5	101	23.4	22.9
RRM12 WT	23.1 ± 0.3	23.9 ± 0.6	79	19.8	18.9
RRM12 K21/F23A	23.0 ± 0.2	23.6 ± 0.3	82	19.8	18.5
RRM12 R27/F40/F43A	22.4 ± 0.2	23.0 ± 0.3	80	19.8	18.5
RRM12 F63/F65A	23.2 ± 0.4	23.7 ± 0.4	81	19.8	19.1
RRM12 K76/R82A	22.6 ± 0.3	23.2 ± 0.5	80	19.8	18.8
xrRNA2-RRM12 complex	29.8 ± 0.1	31.9 ± 0.2	123	43.2	40.2

[a]Derived from Guinier fitting; [b]derived from GNOM analysis; [c]MW: molecular weight predicted from sequences; [d]MW: molecular weight calculated based on the power law of volume of correlation.

Table S2. Thermodynamic parameters of MSI1 RRM12 and its subconstructs or mutants binding to various RNAs at 25 °C.

RNA	Protein	ΔH [kcal/mol]	-TΔS [kcal/mol]	n	Kd [μM]
ZIKV 3'UTR	RRM12	-17.9 ± 0.4	10.1	0.9 ± 0.1	2.5 ± 0.5
ZIKV 3'UTR	RRM1	N.D.			
ZIKV 3'UTR	RRM2	N.D.			
ZIKV 3'UTR pMBS1m	RRM12	-3.3 ± 0.1	0.9	2.9 ± 0.4	253.5 ± 18.1
ZIKV 3'UTR pMBS2m	RRM12	-16.1 ± 0.9	8.9	1.1 ± 0.1	4.7 ± 0.1
ZIKV 3'UTR pMBS3m	RRM12	-17.4 ± 0.7	9.7	1.0 ± 0.1	4.2 ± 0.3
ZIKV xrRNA2	RRM12	-17.8 ± 0.7	10.0	1.1 ± 0.1	1.9 ± 0.8
ZIKV DB12	RRM12	N.D.			
ZIKV 3'SL	RRM12	-6.3 ± 0.9	1.7	1.5 ± 0.5	65.5 ± 0.4
ZIKV xrRNA2-P2M/P4M	RRM12	N.D.			
ZIKV xrRNA2-P2M	RRM12	N.D.			
ZIKV xrRNA2-P4M	RRM12	-18.6 ± 0.2	6.7	0.9 ± 0.1	2.6 ± 0.4
ZIKV xrRNA2-ΔP4	RRM12	-14.9 ± 0.1	5.3	0.9 ± 0.2	5.7 ± 0.1
ZIKV xrRNA2-P2-GAGA	RRM12	N.D.			
ZIKV xrRNA2-P2-GCAA	RRM12	N.D.			
ZIKV xrRNA2-P2-AGCU	RRM12	-14.4 ± 1.2	6.4	1.1 ± 0.1	1.3 ± 0.1
ZIKV xrRNA2-P2-AGUU	RRM12	-12.4 ± 1.7	6.3	1.0 ± 0.1	1.6 ± 0.2
JEV 3'UTR	RRM12	-3.4 ± 0.9	0.3	1.7 ± 0.4	218.0 ± 14.7
USUV 3'UTR	RRM12	-2.8 ± 0.6	0.4	2.2 ± 0.5	301.4 ± 20.5
MVEV 3'UTR	RRM12	-3.0 ± 0.5	0.4	2.0 ± 0.3	264.6 ± 12.5
DENV4 3'UTR	RRM12	-1.2 ± 0.6	0.3	4.6 ± 0.4	335.7 ± 14.8

DENV4 3'UTR mutant	RRM12	-18.82±0.57	10.93	1.29±0.35	4.68±0.74
DENV4 xrRNA	RRM12	N.D.			
DENV4 xrRNA mutant	RRM12	-17.65±0.19	9.24	1.12±0.18	2.54±0.31
ZIKV xrRNA1	RRM12	-5.7 ± 0.7	1.5	1.4 ± 0.3	94.6 ± 1.3
ZIKV xrRNA2	RRM12 K21A/F23A	-15.5 ± 0.5	8.5	0.8 ± 0.3	4.6 ± 0.3
ZIKV xrRNA2	RRM12 R37A/F40A/F43A	-6.5 ± 0.9	1.8	1.8 ± 0.1	99.0 ± 4.0
ZIKV xrRNA2	RRM12 F63A/F65A	-7.4 ± 0.2	2.3	1.8 ± 0.2	38.3 ± 1.9
ZIKV xrRNA2	RRM12 K76A/R82A	-14.1 ± 0.5	6.3	1.00 ± 0.1	6.0 ± 0.7

N.D.: Not detected

Table S3. DNA Primer sets utilized to introduce mutations to RNAs and proteins.

Substitution	Primer name	Sequence（5’-3’）
pMBS1m	mpMBS1-F	ACCAAGCCCAAAGTCAGGCCG
	mpMBS1-R	TTCCCAGCTTCTCCTGGG
xrRNA2 P2M	P2M-F	TAGTCAGGCCCGAAAGGCCATGGCACG
	P2M-R	TGGGCTTGGTTTCCCAGC
xrRNA2 P2-GAGA	P2M2-F	CACTATAGGTAGTCAGGCCCGAGAGGCCATGGCACGGAAGAA
	P2M2-R	CTTCTTCCGTGCCATGGCCTCTCGGGCCTGACTACCTATAGTG
xrRNA2 P2-GCAA	P2M3-F	CACTATAGGTAGTCAGGCCCGCAAGGCCATGGCACGGAAGAA
	P2M3-R	CTTCTTCCGTGCCATGGCCTTGCGGGCCTGACTACCTATAGTG
xrRNA2 P2-AGCU	P2M4-F	CTTCTTCCGTGCCATGGCGAGCTCGGCCTGACTACCTATAGTG
	P2M4-R	CTTCTTCCGTGCCATGGCGAGCTCGGCCTGATTCTACCTATAGTG
xrRNA2 P2-AGUU	P2M5-F	CTTCTTCCGTGCCATGGCGAGTTCGGCCTGACTACCTATAGTG
	P2M5-R	CTTCTTCCGTGCCATGGCGAACTCGGCCTGACTACCTATAGTG
ZIKV 3’UTR PK1	PK1-F	CACGGAAGAAGCCATGGAGCCTGTGAGCCCCTCAGAG
	PK1-R	CTCTGAGGGGCTCACAGGCTCCATGGCTTCTTCCGTG
DENV4 xrRNA Mutant	DENV4M-F	GAGACTATTGTAGTCAGGCCGAGAACGCCACGGCTTGAGC
	DENV4M-R	GCTCAAGCCGTGGCGTTCTCGGCCTGACTACAATAGTCTC
MSI1 K21/F23A	K21/F23A-F	GTTCCAGGGGCCCCTGGGATCCGCGATGGCGATTGGCGGCCTGAGCTGGC
	K21/F23A-R	GCCAGCTCAGGCCGCCAATCGCCATCGCGGATCCCAGGGGCCCCTGGAAC
MSI1 R31A	R31A-F	GACCACCCAGGAAGGTTTAGCGGAATATTTTGGCCAGTTTGG
	R31A-R	GCCAAACTGGCCAAAATATTCCGCTAAACCTTCCTGGGTGGTC
MSI1 F40A	F40A-F	GACCACCCAGGAAGGTTTAGCGGAATATTTTGGCCAGTTTGG

	F40A-R	GCCAAACTGGCCAAAATATTCCGCTAAACCTTCCTGGGTGGTC
MSI1 F43A	F43A-F	ACGCGAATATTTTGGCCAGGCGGGCGAAGTGAAAGAATGCC
	F43A-R	GCCAAACTGGCCAAAATATTCCGCTAAACCTTCCTGGGTGGTC
MSI1 K76A	K76A-F	GGATCAGGCGGGCGTGGATGCGGTGCTGGCGCAAAGCCGTC
	K76A-R	GACGGCTTTGCGCCAGCACCGCATCCACGCCCGCCTGATCC
MSI1 R82A	R82A-F	GATAAAGTGCTGGCGCAAAGCGCGCATGAACTGGATAGCAAA
	R82A-R	GTTTTGCTATCCAGTTCATGCGCGCTTTGCGCCAGCACTTTATC
MSI1 F63/65A	F63/65A-F	CTGACCAAACGCAGCCGTGGTGCGGGCGCGGTGACCTTTATGGATCAGGC
	F63/65A-R	GCCTGATCCATAAAGGTCACCGCGCCCGCACCACGGCTGCGTTTGGTCAG
ZIKV xrRNA2-ΔP4	xrRNA2 ΔP4-R	GGCTCACAGGCAGCATGGCTTCTTC

Table S4. Virus acronym, names and Accession IDs for sequence alignment of MBFV xrRNAs.

Virus	Full name	GenBank accession number
ZIKV	Zika virus	KU955593.1
DENV1	Dengue virus serotype 1	NC_001477.1
DENV2	Dengue virus serotype 2	NC_001474.2
DENV3	Dengue virus serotype 3	NC_001475.2
DENV4	Dengue virus serotype 4	NC_002640.1
BSQV	Bussuquara virus	AY632536.4
SEPV	Sepik virus	DQ837642.1
KOKV	Kokobera virus	NC_009029.2
JEV	Japanese encephalitis virus	NC_001437.1
IGUV	Iguape virus	AY632538.4
ALFV	Alfuy virus	AY898809.1
USUV	Usutu virus	JX473240.1
WNV	West Nile virus	NC_009942.1
KUNV	Kunjin virus	L24512.1
SLEV	Saint Louis encephalitis virus	NC_007580.2
MVEV	Murray Valley encephalitis virus	NC_000943.1
ROCV	Rocio virus	MF461639.1
YFV	Yellow fever virus	NC_002031.1
ILHV	Ilheus virus	KC481679.1
BAGV	Bagaza virus	MF380434.1
BgV	Bamaga virus	MH257544.1
FRV	Fitzroy River Virus	KM361634.1
ITV	Israel turkey meningoencephalomyelitis virus	MN057643.1
KEDV	Kedougou virus	NC_012533.1
KOTV	Koutango virus	MN057643.1
TMUV	Tembusu virus	MN649267.1
WSSV	Wesselsbron virus	NC_012735.1

Table S5. Summary table of HDX-MS experiments.

Data Set	RRM12 alone	RRM12-xrRNA2 complex
HDX reaction details	20 mM HEPES, 100 mM KCl, 3 mM $MgCl_2$ and 0.5 mM TCEP, pD_{read}=7.2	
HDX time course (s)	0, 60, 90, 300	0, 60, 90, 300
HDX control samples	Maximally labeled control (RRM12 alone)	Maximally labeled control (RRM12 alone)
Back-exchange (mean/IQR)	42.05%/14.18%	
# of Peptide	98	102
Sequence coverage	95%	95%
Average peptide length/Redundancy	15.4/8.8	15.3/8.8
Replicates	3 (technical)	3 (technical)
Repeatability	0.0538 (average standard deviation)	0.0624 (average standard deviation)
Significant difference in HDX (delta HDX>X D)	0.273 (99% CI)	

Table S6. Parameters regarding to number of structures and steps for each stage during docking.

index	Description	Parameters#	Default value
1	number of structures for rigid body docking	10000	1000
2	number of structures for refinement/analysis	400	200
3	MD steps during second cooling stage with flexible side-chains at interface	100	100
4	MD steps during third cooling stage with fully flexible interface	1000	1000
5	MD steps for heating phase	200	200
6	MD steps for 300K sampling phase	1250	1250
7	MD steps for cooling phase	500	500

used in our current docking

Table S7. Residue list for Ambiguous Interaction Restraints related to fig S7.

interface	residue type	residue list
RRM12	Active	R37, E38, Q42, F43, K76, L78, A79, Q80, S81, R82, E84, K110, F112, V118, N119, F142, E143, K144, T145, T146, N147, R148, R150, F152
	Passive	Automatically defined
xrRNA2	Active	A2, U3, A4, G13, A14, G15, A16, A17, C18
	Passive	Automatically defined
