# Supplementary material for: Zika virus RNA structure controls its unique neurotropism by bipartite binding to Musashi-1
Source: Nat Commun. 2023 Feb 28;14:1134. doi: 10.1038/s41467-023-36838-w (PMC9972320; doi:10.1038/s41467-023-36838-w)
Supplement: Supplementary file 3 — Description of Additional Supplementary Files [file 41467_2023_36838_MOESM3_ESM.pdf]

Description of Additional Supplementary Files

File Name: Supplementary Data 1
Description: HDX-MS data on peptide coverage and deuteration of RRM12 in the absence and presence of xrRNA2 at three different time points.
