# Supplementary material for: Zika virus RNA structure controls its unique neurotropism by bipartite binding to Musashi-1
Source: Nat Commun. 2023 Feb 28;14:1134. doi: 10.1038/s41467-023-36838-w (PMC9972320; doi:10.1038/s41467-023-36838-w)
Supplement: Supplementary file 5 — Reporting Summary [file 41467_2023_36838_MOESM5_ESM.pdf]

nature portfolio

Corresponding author(s): Cheng-Feng Qin

Last updated by author(s): Feb 2, 2023

Reporting Summary

Nature Portfolio wishes to improve the reproducibility of the work that we publish. This form provides structure for consistency and transparency in reporting. For further information on Nature Portfolio policies, see our Editorial Policies and the Editorial Policy Checklist.

Statistics

For all statistical analyses, confirm that the following items are present in the figure legend, table legend, main text, or Methods section.

n/a	Confirmed	
☐	☒	The exact sample size (*n*) for each experimental group/condition, given as a discrete number and unit of measurement
☐	☒	A statement on whether measurements were taken from distinct samples or whether the same sample was measured repeatedly
☐	☒	The statistical test(s) used AND whether they are one- or two-sided<br>*Only common tests should be described solely by name; describe more complex techniques in the Methods section.*
☒	☐	A description of all covariates tested
☒	☐	A description of any assumptions or corrections, such as tests of normality and adjustment for multiple comparisons
☐	☒	A full description of the statistical parameters including central tendency (e.g. means) or other basic estimates (e.g. regression coefficient) AND variation (e.g. standard deviation) or associated estimates of uncertainty (e.g. confidence intervals)
☐	☒	For null hypothesis testing, the test statistic (e.g. *F*, *t*, *r*) with confidence intervals, effect sizes, degrees of freedom and *P* value noted<br>*Give P values as exact values whenever suitable.*
☒	☐	For Bayesian analysis, information on the choice of priors and Markov chain Monte Carlo settings
☒	☐	For hierarchical and complex designs, identification of the appropriate level for tests and full reporting of outcomes
☐	☒	Estimates of effect sizes (e.g. Cohen's *d*, Pearson's *r*), indicating how they were calculated

Our web collection on statistics for biologists contains articles on many of the points above.

Software and code

Policy information about availability of computer code

Data collection	Pearson's correlation coefficient (PCC) was analyzed by FIJI software with the Coloc2 plugin;<br>maximum likelihood tree was constructed by MEGA (Version 11.0.10);<br>Proteome Discover1.4, HDExaminer for HDX-MS data analysis; MatlabR2017a, BioXTASRAW1.3.1, PRIMUS3.2, GNOM4.6, CRYSOL2.8.3, MONSA1.44 for SAXS data analysis; MODELLER, FARFAR2 and HADDOCK2.4 for structure modeling.
Data analysis	q-RT-PCR/infection rate: The data were analyzed in Microsoft Excel (Version 15.29) and Prism 8 (Version 8.1.2). P-values were calculated byunpaired two-sided t-test. For virus growth curve analysis, multiple comparison was made to the respective control group and p-value was adjusted by Bonferroni correction.<br>Origin 7.0 program for ITC data analysis

For manuscripts utilizing custom algorithms or software that are central to the research but not yet described in published literature, software must be made available to editors and reviewers. We strongly encourage code deposition in a community repository (e.g. GitHub). See the Nature Portfolio guidelines for submitting code & software for further information.

Data

Policy information about availability of data

All manuscripts must include a data availability statement. This statement should provide the following information, where applicable:

- Accession codes, unique identifiers, or web links for publicly available datasets
- A description of any restrictions on data availability
- For clinical datasets or third party data, please ensure that the statement adheres to our policy

The source data underlying Figs. 1f–h, 2a-e, 4a-d, 5d-e, 6c-e and Supplementary Figs. 2c-g, 2j-l, 3g-m, 3i-j, 4b-dare provided as a Source data file. Unprocessed original scans of blots are shown in Source data file. The remaining data are contained within the Supplementary Information or are available from the authors upon request.

Human research participants

Policy information about studies involving human research participants and Sex and Gender in Research.

Reporting on sex and gender	N/A
Population characteristics	N/A
Recruitment	N/A
Ethics oversight	N/A

Note that full information on the approval of the study protocol must also be provided in the manuscript.

Field-specific reporting

Please select the one below that is the best fit for your research. If you are not sure, read the appropriate sections before making your selection.

☒ Life sciences ☐ Behavioural & social sciences ☐ Ecological, evolutionary & environmental sciences

For a reference copy of the document with all sections, see nature.com/documents/nr-reporting-summary-flat.pdf

Life sciences study design

All studies must disclose on these points even when the disclosure is negative.

Sample size	Sample size was estimated on the basis of similar research reported in the literature.
Data exclusions	No data were excluded from analyses
Replication	All experiments were performed using at least 3 biological replicates to ensure reproducibility.
Randomization	All samples were analyzed equally with no subsampling. Therefore, there was no requirement for randomization.
Blinding	Investigators were generally not blinded as the experimental conditions required investigators to know the identity of the samples.

Reporting for specific materials, systems and methods

We require information from authors about some types of materials, experimental systems and methods used in many studies. Here, indicate whether each material, system or method listed is relevant to your study. If you are not sure if a list item applies to your research, read the appropriate section before selecting a response.

Materials & experimental systems

n/a	Involved in the study
☐	☒ Antibodies
☐	☒ Eukaryotic cell lines
☒	☐ Palaeontology and archaeology
☒	☐ Animals and other organisms
☒	☐ Clinical data
☒	☐ Dual use research of concern

Methods

n/a	Involved in the study
☒	☐ ChIP-seq
☒	☐ Flow cytometry
☒	☐ MRI-based neuroimaging

Antibodies

Antibodies used

Antibody	Species	Lot. No	Supplier	Catalog No
anti-FMRP	Rabbit	4000001026	ABclonal	A4539
anti-MSI1	Rabbit	GR3259783-1	Abcam	ab97959
anti-ZIKV E	Mouse		Biofront technologies	BF-1176-46
anti-DENV E	Mouse		produced in our lab	
anti-Sox2	Rabbit	GR3186553-4	Abcam	ab97959
anti-Actin	Rabbit	9100026001	ABclonal	AC026
anti-Flag	Rabbit		Sigma Aldrich	F1804
anti-dsRNA	mouse	J2-2107	Scicons	1010200
mouse IgG isotype control	Mouse	GR3410700-2	Abcam	ab37355
goat anti-rabbit IgG (alexa fluor 594)	goat	GR3323881-1	Abcam	ab150080
goat anti-mouse IgG (alexa fluor 488)	goat	GR3353661-2	Abcam	ab150113
hrp-conjugated goat anti-rabbit IgG	goat	205000819	ZSGB-BIO	ZB-2301
hrp-conjugated goat anti-mouse IgG	goat	203700825	ZSGB-BIO	ZB-2305

Validation

All Antibodies were validated by the manufacturer.
anti-FMRP We confirmed that the FMRP band at ~75 kDa in BHK-21 cells by western blot.
anti-MSI1 We validated the MSI1 band at ~38 kDa in NPC, SH-SY5Y, U251 cells and BHK-21 cells with MSI1 overexpression .We validated it by western blot in NPC cells with MSI1 knockdown. We observed specific fluorescence in SH-SY5Y, U251 cells and BHK-21 cells with MSI1 overexpression in indirect immunofluorescence assay.
anti-ZIKV E We validated the ZIKV E protein band at ~55 kDa in NPC cells infected with ZIKV. We observed specific fluorescence in in NPC, SH-SY5Y, U251 and BHK-21 cells infected with ZIKV.
anti-DENV E We observed specific fluorescence in NPC and BHK-21 cells infected with DENV.
anti-Sox2 We observed specific fluorescence in NPC cells.
anti-Actin We validated a single band at ~45 kDa in different cell lines by western blot.
anti-Flag We validated it by observing correct molecular weight in western blot analysis for Flag-MSI1 protein. We also tested Flag Ab by IP proteins tagged with Flag and confirmed in Western blot analysis.
anti-dsRNA We observed specific fluorescence in BHK-21 cells infected with ZIKV.

Eukaryotic cell lines

Policy information about cell lines and Sex and Gender in Research

Cell line source(s)

BHK-21 ATCC CCL10 baby hamster kidney cell line
Vero ATCC CCL81 monkey kidney cell line
SH-SY5Y ATCC CRL-2266 human neuroblastoma cell line
U251 NICR 1101HUM-PUMC000058 human glioblastoma cell line
hNPC line 15167 human neuroprogenitor cell line provided by S. Bao (Cleveland Clinic)
C6/36 ATCC CRL-1660 Aedes albopictus cell line

Authentication

Cells were authenticated by comparing them to the original morphological and growth characteristics and were verified using short tandem repeat (STR) markers.

Mycoplasma contamination

We confirmed that all cell lines were tested negative for mycoplasma contamination.

Commonly misidentified lines
(See ICLAC register)

None.
